# Phased Patagonian Ice Sheet response to Southern Hemisphere atmospheric and oceanic warming between 18 and 17 ka

**DOI:** 10.1038/s41598-019-39750-w

**Published:** 2019-03-11

**Authors:** Jacob M. Bendle, Adrian P. Palmer, Varyl R. Thorndycraft, Ian P. Matthews

**Affiliations:** Centre for Quaternary Research, Geography Department, Royal Holloway, University of London, Egham, TW20 0EX Surrey, UK

## Abstract

The onset of deglaciation in the Southern Hemisphere mid-latitudes has been attributed to the southward transmission of climate anomalies in response to slow-down of Atlantic meridional overturning circulation (AMOC) during Heinrich Stadial 1 (HS-1; 18–14.6 ka). However, inferences on the response of former ice sheets to sub-millennial palaeoclimate shifts are limited by a shortage of high-resolution terrestrial archives. Here we use a ~1000-year duration, annually-resolved lake sediment record to investigate the deglacial retreat dynamics of the Lago General Carrera–Buenos Aires ice lobe (46.5°S) of the former Patagonian Ice Sheet. We attribute the onset of glacier retreat at 18.0 ± 0.14 cal ka BP to abrupt southward migration of the Southern Westerly Winds that enhanced solar radiation receipt (and ablation) at the ice sheet surface. We infer that accelerated retreat from 17.77 ± 0.13 cal ka BP represents a lagged Southern Hemisphere response to gradual ocean-atmosphere warming associated with the centennial-scale transmission of Northern Hemisphere climate anomalies through the oceanic bipolar seesaw. By 17.38 ± 0.12 cal ka BP, the glacier margin had receded into a deepening proglacial lake, instigating sustained calving losses and more rapid ice recession.

## Introduction

The mid-latitude ice masses of Patagonia and New Zealand are ideally located to investigate the response of the cryosphere to climatic changes that occurred during the global Last Glacial Maximum (LGM) and succeeding deglacial period^1–3^. In particular, the southern mid-latitude region is strongly influenced by variations in the strength and position of the Southern Westerly Winds (SWWs)^4,5^ and Southern Ocean fronts^6–8^. Physical changes in this coupled system play an important role in major climate transitions, influencing ocean circulation^9^ and the global carbon cycle^10^, and transmitting climate anomalies between regions, on timescales of centuries to decades^11–13^. Constraining the dynamics of former mid-latitude ice sheets through such transitions therefore offers important insight into cryosphere response to regional and hemispheric climate drivers^14^. However, owing to the chronological uncertainties associated with radiometric dating of former glacier margins, chronologies of ice recession often overlap intervals of rapid climate transition^1,3,15–17^. Whether former mid-latitude ice sheets bear the imprint of (sub-)centennial, as well as millennial-scale climate variability, therefore, remains uncertain, precluding empirical assessments of (i) the intra- and interhemispheric phasing of palaeoclimate between the poles^18–20^ and mid-latitudes; and (ii) theoretical^21,22^ and simulated^11,23^ models of climate reorganisation during glacial-to-interglacial transitions. Incremental dating techniques, however, such as annual-layer counting of varved lake sediment sequences, offer palaeoenvironmental data with the potential to resolve sub-centennial climate variability (e.g. ref.^24^).

## The FCMC17 varve record

**Figure 1:**
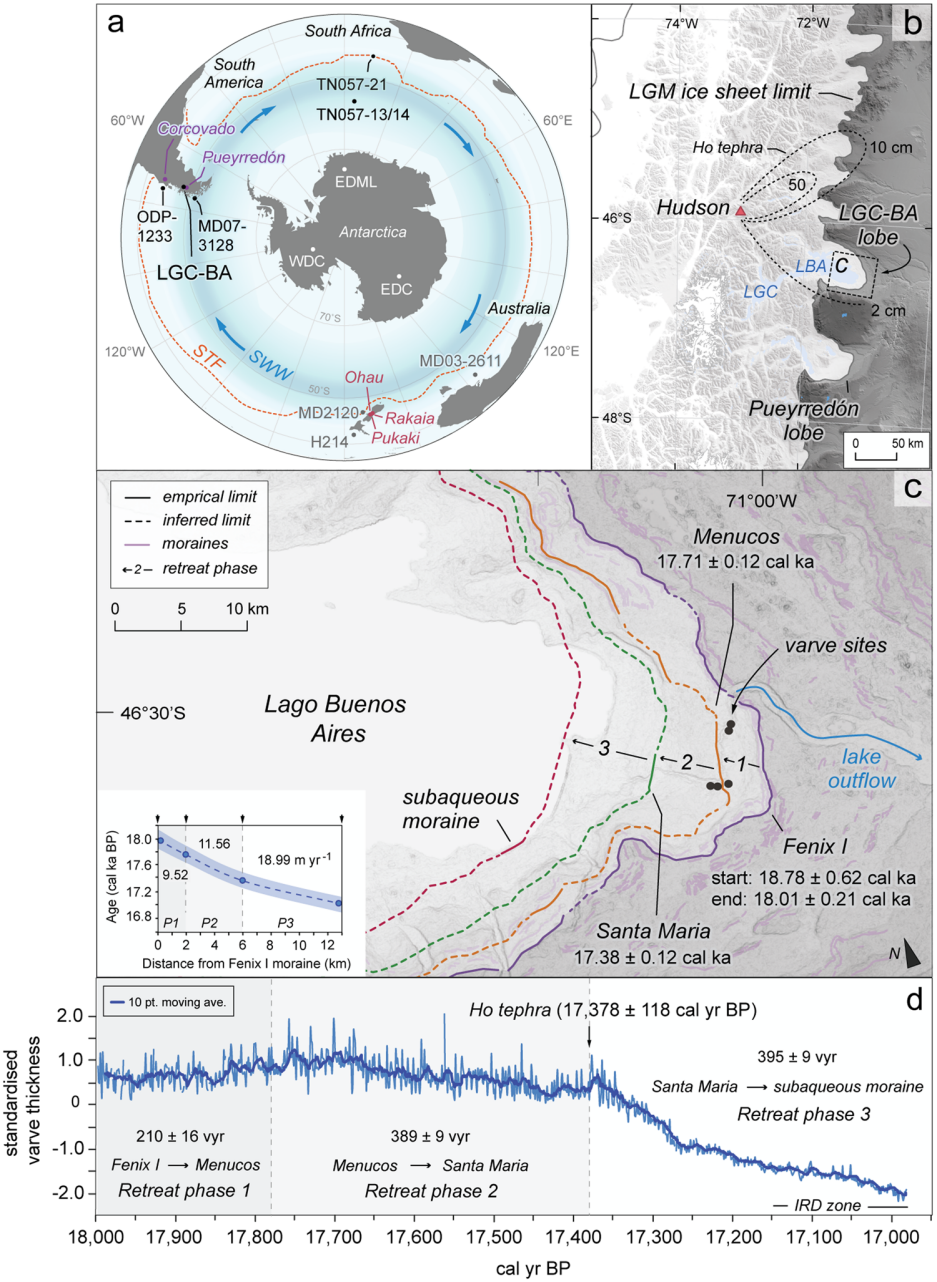
Study location and the FCMC17 varve record. (**a**) Location of LGC–BA, and other Southern Hemisphere palaeoclimate archives, with contemporary position of the Subtropical Front (STF)^63^ and Southern Westerly Wind (SWW) system^1^ indicated. Records labeled in grey are referenced in the text, but not shown on Fig. 3 (MD2120 and H21436; MD03-2611^64^). (**b**) LGM limits of the PIS in central Patagonia^65^. (**c**) Ice-lobe retreat phases and ice margins (including modelled ages of moraine deposition; cf. ref.^25^). Inset: time-distance path of LGC–BA ice-lobe retreat. The varve chronology error is indicated with the blue envelope. (**d**) FCMC17 standardised varve thickness record (reformatted from published figure in ref.^25^), a proxy for sediment flux, with retreat phases 1–3 shown.

**Figure 2:**
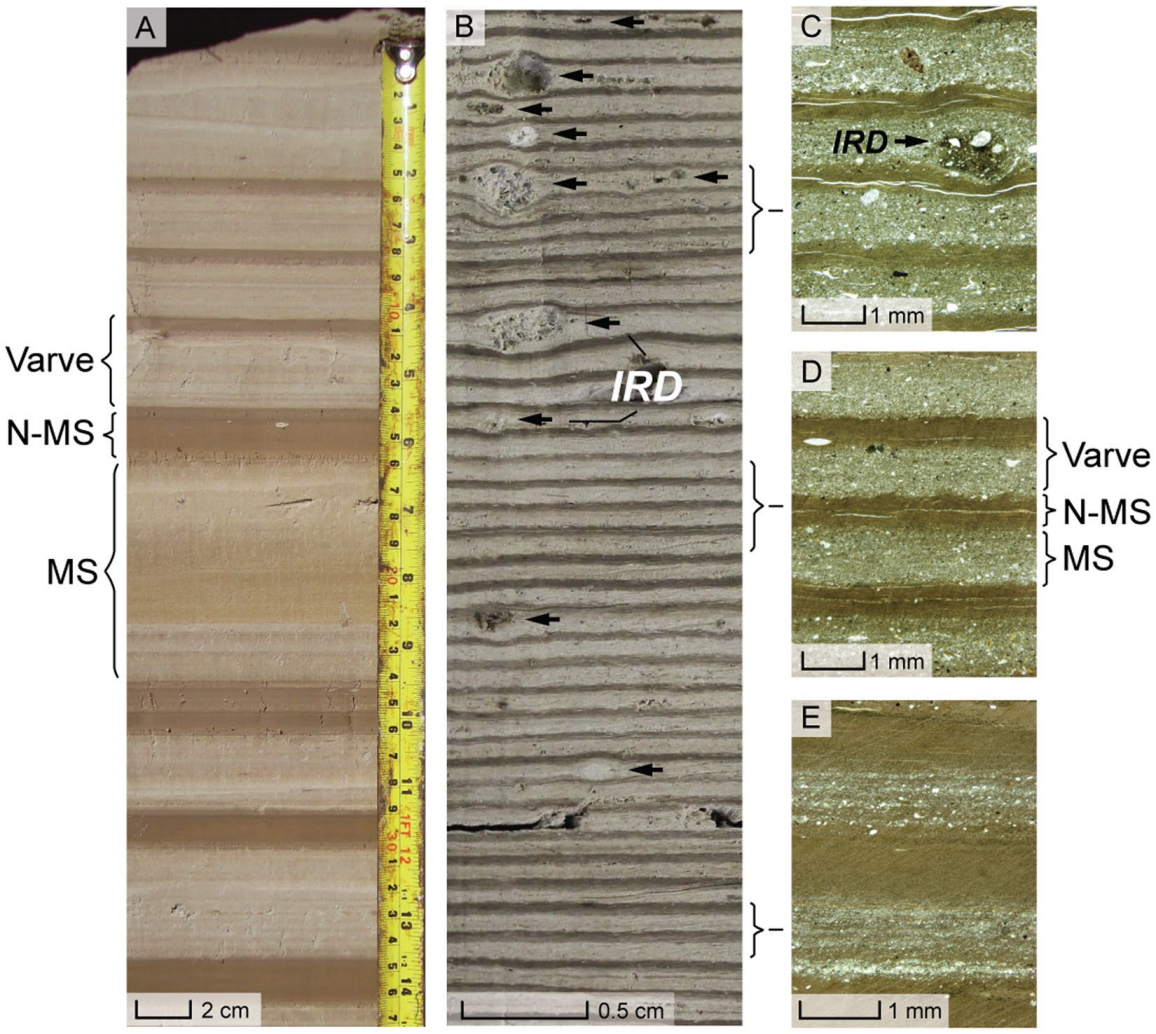
FCMC17 varved sediments. (**A**) Thick (>1 cm) varves deposited during Retreat Phase 2 (see Fig. 1). (**B**) Thin (mm) varves deposited during Retreat Phase 3, with thin-section photo-micrographs showing interval varve structure (**C**–**E**). Note the onset of ice-rafted debris (IRD) deposition in (**C**). MS: melt season layer; N-MS: non-melt season layer.

Here, we report a glaciolacustrine varve record (see Supplementary Information) of outlet glacier retreat dynamics from the Lago General Carrera (Chile)–Buenos Aires (Argentina) basin (LGC–BA; Fig. 1)^25^, the largest conduit of former Patagonian Ice Sheet (PIS) discharge in central Patagonia (46′34°S; 71′03°W)^26^. The 994 ± 36 varve year (vyr) record is anchored to the calendar-year timescale through the presence of the precisely-dated Ho tephra from Cerro Hudson (17,378 ± 118 cal yr BP; see Supplementary Information) and records the initial stages (~18–17 ka) of PIS deglaciation at 46.5°S^25^. Glaciolacustrine varved sediments accumulated in an ice-contact proglacial lake that formed and expanded as the LGC–BA ice lobe retreated from the local LGM terminal ice limit^25^. Through detailed sedimentological investigation of these sequences, Bendle *et al*. (ref.^25^) developed the Fenix Chico Master Varve Chronology (FCMC17), a composite record derived from five varve series located in the eastern parts of the LGC–BA basin. The FCMC17 varves are composed of two main sedimentary layers (Fig. 2): a silt and/or fine sand layer deposited during the melt season (spring/summer) in response to glacier ablation and sediment transport through the glaciohydraulic system; and a clay layer that settled from suspension during the quiescent non-melt season (autumn/winter) when glacier ablation ceased, and meltwater/sediment fluxes to the lake diminished^25^. Together, these two layers form a varve couplet representing one year of sedimentation^25,27,28^.

The thickness of annual varve couplets are a proxy of past glacial activity in LGC–BA^25^. In ice-contact glacial lakes, the flux of glaciolacustrine sediment is controlled by the capacity of the glacial meltwater system in transporting stored erosion products to the lake^28–31^. Meltwater production rates are strongly linked to climate conditions during the spring/summer melt season, with the strongest correlations in modern lake systems observed between varve thickness and air temperature (e.g. refs^29–35^). Over multi-decadal to centennial timescales, meltwater and sediment fluxes are modulated by glacial extent, where a more proximal (distal) ice margin leads to increased (decreased) sediment flux and varve thickness^28,31–33,36^. Over decadal scales, the controls on varve thickness are more complex. Increased varve thickness has been linked to transitional periods of (i) rapid glacier advance (i.e. increased ice-margin proximity) or (ii) the initial phases (one-to-two decades) of glacier retreat (i.e. enhanced glacier ablation and meltwater/sediment flux)^31,36^. Using these established glacier-varve relationships, high-resolution (<centennial) analyses of glaciolacustrine sediment flux (i.e. varve thickness) are related to changes in former ice-margin position (Fig. 1c) to calculate the mean retreat rate of the LGC–BA ice lobe at the onset of regional deglaciation (see Methods).

### Decadally-resolved retreat dynamics of the LGC–BA ice lobe

Prior to retreat, the LGC-BA ice lobe remained stable at the local Last Glacial Maximum (LGM) Fenix I moraine for ~700 modelled years, between 18,778 ± 615 and 18,086 ± 214 cal yr BP (Fig. 1c)^25^. Upon retreat, glacial lake expansion allowed varves to form. Three major phases of ice lobe recession are observed in the FCMC17 record (see Fig. 1d; reformatted from published figure in ref.^25^).

Phase 1 starts at 17,997 ± 144 cal yr BP (hereafter 18.0 ± 0.14 cal ka) and represents a ~2 km terminus retreat from the Fenix I to Menucos moraine (Fig. 1c). We identified a minimum of 210 ± 16 varves between these former ice limits, yielding a mean ice retreat rate of ~9.5 m yr^−1^ As we were unable to identify ‘basal varves’ (i.e. varves directly overlying glacial till) in this area, the calculated retreat rate may be an overestimate. However, the very thick varves observed in these sequences are typical of ice-proximal sedimentation during the earliest decades of ice-retreat^25,28^, likely making this overestimate minor (Fig. 1d).

The onset of varve deposition behind the Menucos moraine marks the start of Phase 2, which is characterised by an overall decrease in mean varve thickness over a minimum of 389 ± 9 vyrs. The retreat from the Menucos moraine begins abruptly, with a short-lived (10 year) increase in varve thickness from 17,768 ± 127 cal yr BP (hereafter 17.77 ± 0.13 cal ka; Fig. 1). In other systems, comparable short-lived increases in glaciolacustrine sediment flux have been interpreted to represent enhanced ablation and meltwater production during the initial decades of ice-margin retreat^31,36^. We also invoke this interpretation, which would suggest that varves started to accumulate almost immediately behind the ice-contact face of the Menucos moraine. The subsequent decrease in varve thickness through the rest of Phase 2 (Fig. 1d) documents accelerated ice-margin retreat (~21% faster than during Phase 1) to the Santa Maria ice-contact fan (Fig. 1c), which represents a minimum ice-retreat distance for this phase (see Supplementary Information). The ~4.5 km ice-margin recession from the Menucos moraine to the Santa Maria ice-contact fan yields a mean ice-retreat rate of ~11.6 m yr^−1^ (Fig. 1).

**Figure 3:**
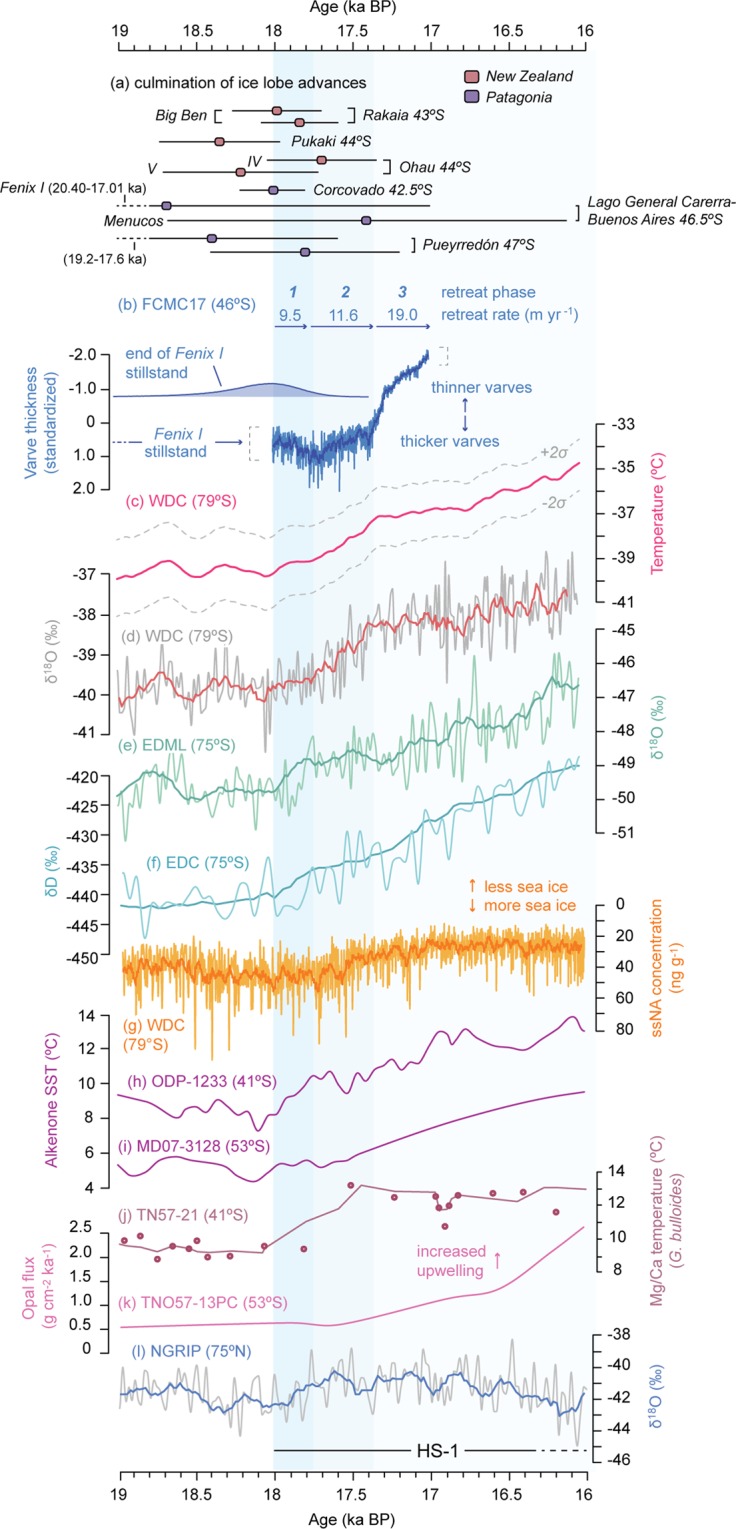
Comparison of the FCMC17 ice-lobe retreat record with Southern Hemisphere palaeoclimate archives. (**a**) The timing of final ice-lobe advances from selected sites in Patagonia^15,66–68^ and New Zealand^3,54^. (**b**) LGC–BA varve thickness and ice-lobe retreat rates. Modelled end of Fenix I stability from ref.^25^. (**c**) West Antarctic temperature reconstruction from WDC ice-core^38^. (**d**) WDC δ^18^O (temperature) record^18^. WDC records shown on WD2014 timescale^69^. (**e**) East Antarctic δ^18^O (temperature) record from the EDML ice-core^18^. (**f**) East Antarctic δD (temperature) record from the EDC ice-core^39^. EDML and EDC shown on Limeux-Dudon *et al*. (ref.^70^) timescale. (**g**) WDC ssNA record^19^. Alkenone-derived SST reconstruction from (**h**) ODP-1233^6^, and (**i**) MD07-3128^42^. ODP-1233 and MD07-3128 use age models from ref.^50^. (**j**) Mg/Ca temperature reconstruction from core TN57-21^7^. (**k**) Opal flux record from core TN057-13^43^. (**l**) NGRIP δ^18^O record^31^ of Greenland temperature on GICC05 timescale^71^.

Phase 3, which lasted a minimum of 395 ± 9 vyrs, starts at 17,378 ± 118 cal yr BP (hereafter 17.38 ± 0.12 cal ka) with a 123 ± 3 vyr period of abruptly decreasing varve thickness. Subsequently, the rate of varve thickness decrease slows at 17,255 ± 122 cal yr BP and remains relatively constant until the end of the FCMC17 record at 16,982 ± 127 cal yr BP (hereafter 16.98 ± 0.13 cal ka; Fig. 3b). We observe a persistent increase in the volume of ice-rafted debris (IRD; Fig. 2B) at 17,145 ± 122 cal yr BP (Fig. 1d), suggesting that a permanent calving ice-front had developed by this time^37^ and that the LGC-BA ice lobe had retreated into deeper lake waters^25^. The precise ice-margin position(s) established during Phase 3 cannot yet be definitively confirmed. However, a subaqueous moraine complex on the southern shoreline of LGC–BA (see Supplementary Information) provides a minimum constraint on the distance (6.0–9.0 km) of ice recession during Phase 3, which yields a mean ice-margin retreat rate of ~19.0 m yr^−1^ (Fig. 1), 64% faster than during Phase 2.

### Phasing of Southern Hemisphere cryosphere and ocean-atmosphere dynamics

The FCMC17 varve record provides the first mid-latitude terrestrial archive with the capacity to evaluate the sub-millennial phasing of cryosphere and climate dynamics. We assess oceanic and atmospheric controls on regional PIS deglaciation through a 3000-year window between 19.0 and 16.0 ka (Fig. 3). The selected palaeoclimate records offer the highest available temporal resolution from marine and terrestrial environments. Between 19.0 and 18.0 ka, the West Antarctic Ice-Sheet Divide ice-core (WDC) record^18,19^ exhibits relatively stable δ^18^O (annual-mean surface air temperatures) values (Fig. 3d) and reconstructed temperatures (Fig. 3c)^38^ that coincide with the ~700 year period of late-LGM glacier stability at LGC-BA. From 18.0 ka there is a two-step warming at WDC: an initial increase of ~0.5 °C between 18.0–17.8 ka followed by accelerated warming of ~2 °C between 17.8–17.4 ka (Fig. 3c,d). These warming steps are synchronised (within dating uncertainties) with the onset (Phase 1) and acceleration (Phase 2) of ice lobe retreat recorded in the FCMC17 varve record (Fig. 3b). Abrupt atmospheric warming is also recorded in East Antarctic ice cores at 18.0 ka (Fig. 3e,f)^20,39^. The accelerated West Antarctic warming at 17.8 ka also coincides with Southern Ocean sea ice retreat, as indicated by a decrease in sea-salt sodium (ssNa) concentration in the WDC record (Fig. 3g)^18,19^. After 17.4 ka there is a slower rate of West Antarctic warming (Fig. 3c,d) and sea-ice decline (Fig. 3g) yet FCMC17 varve thickness decreases rapidly during Phase 3 (Fig. 3b), demonstrating enhanced ice-margin retreat from 17.38 ± 0.12 cal ka. To explain this divergence, we infer that calving dynamics augmented atmospheric warming as a major control on ice lobe recession. This interpretation is supported by the sustained presence of IRD in the FCMC17 record after 17,145 ± 122 cal yr BP (Fig. 2B)^25^, which suggests that a permanent calving ice-front had established by this time, most likely due to ice-margin retreat into an ice-contact lake that was both expanding laterally and deepening^25^. Such situations have been shown to enhance negative glacier mass balance and accelerated ice retreat^40^, which can at least partially decouple a freshwater calving glacier from the prevailing climate conditions^41^. Thus, we argue that the Phase 3 varve thickness data reflects a combination of climatic and internal glaciological influence on ice-margin retreat rate in LGC–BA.

Over the duration covered by the FCMC17 record, marine sediment cores from the Chilean margin at 41°S (Fig. 3h) and 53°S (Fig. 3i) record Pacific sea-surface temperature (SST) changes north and south of LGC–BA. At 41°S (ODP-1233)^6^, SSTs increase from ~7.5 °C to ~10 °C between 18.1–17.6 ka, overlapping Phase 1 of the FCMC17 record, before more consistent temperatures are attained between ~10 and 12 °C after 17.6 ka. At 53°S (MD07-3128)^42^, SSTs of ~5–6 °C begin to increase at 18.2 ka, before a further 3–4 °C of warming through the rest of the time-window. Palaeoecological temperature reconstructions from TN57-21 in the Southeast Atlantic^7^ support a comparable subsurface warming of ~9–13 °C between 18.1 and 17.5 ka (Fig. 3j). At site TN057-13 in the Southern Ocean (Fig. 3k), opal fluxes rise gradually from 19.0 ka, but increase markedly at 17.7 ka, coinciding with Phase 2 of FCMC17, interpreted as enhanced Southern Ocean upwelling^43^. Within the time-window covered by climate fluctuations in Southern Hemisphere palaeorecords, cool and broadly consistent temperatures are evident in the North Atlantic NGRIP ice-core record (Fig. 3l)^44^, and bracket the period associated with North Atlantic sea-surface cooling and stratification in HS-1^45,46^.

The FCMC17 record demonstrates that the onset (18.0 ± 0.14 ka) and acceleration (17.77 ± 0.13 ka) of PIS ice lobe retreat at 46.5°S (Fig. 3b) were phase-locked with shifts in West Antarctic temperature^18,19^ between ~18.0 and 17.4 ka (Fig. 3c,d), highlighting the potential synchronicity (within dating uncertainties) in atmospheric warming trends over 35° of latitude (between 46.5°S and 80°S), coincident with the broad onset of mid-latitude deglaciation on either side of the Pacific Ocean (Fig. 3a,b)^2,3,15–17^. These changes also overlap the onset of reduced AMOC^45^, and North Atlantic ocean^46,47^ and atmosphere (Fig. 3l) cooling^44^, at the onset of HS-1. This allows us to compare Southern Hemisphere mid-latitude glacier response (Fig. 3b) to interhemispheric palaeoclimate at high temporal resolution.

### Onset of PIS retreat: southward migration of the SWW and STF

First, we consider mechanisms for synchronous onset of WDC warming (Fig. 3c,d) and mid-latitude ice sheet retreat (Fig. 3b) at 18.0 ka. A common feature of the simulated response to perturbed AMOC is a poleward contraction and/or strengthening of the southern westerlies, caused by a southward shift of the Intertropical Convergence Zone (ITCZ)^11,12,23,48^. Marine SST records from the South Atlantic^7^, Southeast Pacific^6,42^, and Southwest Pacific^8,49^ record a concurrent poleward migration of the Subtropical Front (STF), and a strengthening of ACC flow through the Drake Passage^50^, consistent with southward-shifted westerlies. A sustained poleward shift of westerly circulation provides a likely trigger for mid-latitude glacier retreat, as southward-shifted storms tracks would decrease cloud cover over Patagonia and enhance solar radiation receipt^51^ at the ice sheet surface, increasing melting and (together with reduced accumulation) driving negative mass balance. Conceptually, this process is similar to a positive state in the Southern Annular Mode (the leading mode of tropospheric circulation variability south of 20°S), whereby high mid-latitude surface pressure forces a poleward contraction of the SWWs^52^ and increased surface air temperature south of 40°S, due to a combination of horizontal advection, subsidence and enhanced solar radiation^53^. The broadly synchronous timing of deglaciation in New Zealand (Fig. 3a)^3,54^ is consistent with the proposed hemispheric scale of a coupled ocean-atmosphere shift in the SWWs and STF^55^.

To explain the synchronous onset of atmospheric warming in WDC and ice-lobe retreat at LGC–BA around 18.0 ka (Fig. 3d), the initial spread of warmth to the Antarctic continent must have been rapid. Model outputs suggest that SWW shifts can rapidly (in decades) transmit climate anomalies poleward^11,12,40^, a feature confirmed by WDC ice-core deuterium excess records that reveal abrupt poleward moisture source shifts at the onset of Northern Hemisphere stadials^13,55^. However, the modelled southern high-latitude air-temperature response to poleward-shifted westerlies is equivocal, with some models showing warming of ≥1 °C in West Antarctica^11,12^, whereas others display limited temperature change^56^.

### Accelerated PIS retreat: the oceanic bipolar seesaw, sea-ice retreat, and Southern Ocean convection

Second, we consider the processes driving synchronous acceleration of WDC warming (Fig. 3c,d) and mid-latitude glacier retreat (Fig. 3b) at ~17.8 ka, ~200 years after the onset of ice-lobe retreat at LGC–BA (Fig. 3b). Recent model simulations^23^ suggest a lagged southern warming response to the oceanic bipolar seesaw following AMOC collapse in the North Atlantic. The delay in Pacific sector warming reflects the time taken (a century or more) for heat to accumulate in the South Atlantic, and for warm anomalies to be propagated around the Southern Ocean via Kelvin and Rossby waves, or with the ACC flow^23^. This produces modelled Pacific sector warm anomalies of ~1–1.5 °C, both north and south of the ACC, within 100–200 years of AMOC collapse, and with a synchronous surface air-temperature increase of ~1 °C in both Patagonia and Antarctica^23^. 500 years after AMOC collapse, a mid- and high-latitude warming of ~2 °C is simulated, sufficient to explain around half the glaciologically-modelled (summer) atmospheric warming (~4.5 °C) that drove mountain glacier retreat in the Southern Alps of New Zealand during HS-1^3^. This modelled process is empirically supported by analyses of isotopic changes recorded in time-synchronised polar ice cores^55^, which evidence a consistent ~200-year lag between Northern Hemisphere climate and Antarctic temperature response owing to the timescales of anomaly transmission through the oceanic bipolar seesaw.

The spread of heat around the Indian and Pacific Oceans would also steepen the temperature gradient across the ACC, enabling eddy heat fluxes to cross this dynamic physical boundary more readily^23^. Together with a wind-driven southward shift of the STF^6,7^, this process would deliver warm waters to the sea ice zone, driving sea ice retreat and meridional atmospheric heat fluxes both poleward (>70°S) over Antarctica, and equatorward (<50°S) to the mid-latitudes^23^. Southern Ocean deep convection may have further amplified both mid- and high-latitude warming through heat release from intermediate depths^51,57^, forced by either wind-driven upwelling (Fig. 3k)^43^ and/or sea-ice retreat (Fig. 3g)^23^. These processes could have operated independently, or in unison, given that a poleward contraction of the SWW is necessary in both cases. In either situation, within a century of onset, modelled Southern Ocean convection events drive air-temperature increase of ~1 °C in the mid-latitudes, and ~2 °C in Antarctica^57^. The empirical evidence for a phase-locked change to accelerated West Antarctic warming (Fig. 3d)^18,19^, sea-ice retreat (Fig. 3g)^19^, and mid-latitude deglaciation (Fig. 3b) at 17.8 ka, supports the occurrence of these modelled processes.

### PIS response to phased atmospheric and oceanic change

From the above synthesis, we infer that the LGC–BA ice lobe, a major outlet glacier of the former PIS in mid-latitude South America, responded dynamically to a multi-phase sequence of atmosphere (rapid) and ocean (lagged) change during HS-1^23,55^. After the final stages of the local LGM (18,778 ± 615 to 18,086 ± 214 cal yr BP)^25^, the PIS at 46.5°S responded rapidly to interlocked southward migration of the SWWs and STF at ~18.0 ka^6,7,15^. This circulation shift, which modulated the mid-latitude glaciological surface energy budget, regional atmospheric temperature, and moisture distribution, was likely zonally symmetric, as mountain glaciers in New Zealand also started to recede at ~18.0 ka (Fig. 3b)^3,54^. Though our data suggest that the LGC–BA ice lobe responded rapidly (perhaps within decades) to southward-shifted westerlies, the rate of retreat was steady for ~200 years (Fig. 3b). We propose that accelerated glacier retreat after ~17.8 ka reflects a response to lagged oceanic heat transfer from the Atlantic to Pacific Ocean sector^23,55^, the consequent ambient air-temperature increase^23^, and ocean-to-atmosphere heat fluxes associated with processes operating in the Southern Ocean sea-ice zone^23,57^. We speculate, therefore, that southward migration of global wind belts, comprising the low-latitude ITCZ and mid-latitude SWW^3,22,48^, initiated the retreat of Patagonian glaciers at the end of the last glaciation^15–17^. Subsequent ice sheet instability was driven largely by sustained and broadly synchronous warming of the Pacific and Southern Oceans, propagated through the oceanic bipolar seesaw^21,23^. We propose that the cumulative ambient air temperature increase from ~18 ka forced the LGC–BA terminus into deeper lake water, after which calving dynamics contributed significantly to local ice sheet collapse, in addition to background climate warming.

## Conclusion

We have presented an annually-resolved terrestrial archive from the Southern Hemisphere mid-latitude region that, for the first time, allows exploration of the phasing of mid-latitude ice-sheet dynamics and hemispheric palaeoclimate at the end of the last glaciation (~18–17 ka). Our results reveal close synchronicity between regional PIS retreat dynamics and West Antarctic warming and sea-ice trends^18,19^ between ~18.0 and 17.4 ka. Specifically, the timing of major shifts in outlet glacier retreat, inferred from the FCMC17 varve thickness record, support both modelled^23^ and empirical^55^ data in favour of a sequence of interhemispheric ocean-atmosphere changes driven by perturbed AMOC in HS-1. The high-precision and incremental FCMC17 record^25^ shows a phased ice-sheet response to (i) rapid (decadal) coupled migration of the SWW and STF at 18.0 ± 0.13 cal ka; (ii) lagged (centennial) ocean–atmosphere warming from 17.77 ± 0.13 cal ka, transmitted through the oceanic bipolar seesaw, and (iii) a combination of continued warming and local calving dynamics from 17.38 ± 0.12 cal ka.

## Methods

### Production of varve chronology and thickness record

This study was carried out on a composite varve thickness record and chronology (FCMC17) established from five individual site varve records in the Río Fenix Chico valley, at the eastern end of LGC–BA^25^. Varve counts and thickness measurements were generated from field (macroscopic) outcrops (varves >1 cm thickness; 85.35% of composite) or microscopic analyses of petrographic thin sections (varves <1 cm thickness; 14.65% of composite). Site records were correlated on a varve-to-varve basis using prominent marker layers as definitive tie-points between sequences, alongside distinctive trends in varve thickness. Varve count uncertainties were estimated through repeat measurements, although local disturbances were often bridged by better preserved sequences during the correlation process. This process yielded a composite chronology of 994 ± 36 varve years (vyrs)^25^.

### Dating of composite varve record

A prominent time marker was established at vyr 605 ± 27 in the composite record, through geochemical fingerprinting of a visible tephra layer (see Supplementary Information)^25^. Samples from three sites were sieved at 125 µm and 15 µm, and an optimum glass fraction extracted using sodium polytungstate (SPT) at 2.55 g cm^−3^ density^58^. Glass shards were mounted in probe stubs, carbon coated, and major element analyses obtained for 195 individual glass shards using a Cameca-SX100 WDS-EPMA at the Tephra Analytical Unit, University of Edinburgh. Analyses were undertaken using a beam diameter of 5 μm and at 15 keV; a 2 nA beam current was employed for Na, Al, Si, Fe, K, Ca, Mg and a 80 nA beam current for Mn, Cl and Ti^59^. The uncertainty on this analysis is <1%, and international standards Lipari 1 (rhyolitic) and BCR2G (basaltic) were analysed frequently to monitor for instrument drift. In addition, five bulk tephra samples (~10–12 g) were prepared for trace element analyses. Samples were sieved at 15 µm to remove the fine silt and clay fraction, and powdered in a tungsten carbide mill. Powered samples were pressed into pellets and analysed using a PANalytical Axios Sequential X-ray Fluorescence Spectrometer at Royal Holloway, University of London. Matrix corrections were applied using the major element chemical dataset obtained using the WDS-EMPA analysis, and Limits of Detection (LoD) were calculated using long-term data collected using the same equipment^25^. Geochemical correlations with published tephrochronological datasets determined an origin as the Ho tephra from Cerro Hudson^60^, and eight published radiocarbon determinations^61^ were remodeled in Oxcal v4.3^62^ to 17,378 ± 118 cal yr BP. This age was inserted at vyr 605 ± 27 and calendar years extrapolated for the remaining varves. Varve counting errors were propagated backwards and forwards from vyr 605 and summed with the uncertainty of the Ho tephra age^25^.

### Calculation of ice-lobe retreat rates

The number of varves deposited between geomorphologically (i.e. moraines) or stratigraphically defined ice limits enabled calculation of average ice-lobe retreat rates (m yr^−1^) for three main recessional phases. These phases are also evident as prominent changes in the trend of varve thickness. The number of varves deposited between former ice-margin positions was deduced from site varve records, which were strategically located with respect to temporary ice lobe positions (see Supplementary Information). The age-estimate uncertainty associated with the Ho tephra (17,378 ± 118 cal yr BP) permitted absolute dating of ice-lobe retreat dynamics at centennial resolution. However, the continuous FCMC17, with low internal varve count uncertainty (994 ± 36 vyr), enabled the characterisation of former ice-dynamics at centennial, and even decadal, resolution, and permitted comparison with other high-resolution incremental climate records (e.g. refs^18,19^).

## Supplementary information

Supplementary Information

## Data Availability

The FCMC17 varve and tephrochronological data are available at Figshare with the identifier 10.17637/rh.6870908.v1.
