# Supplementary material for: Phased Patagonian Ice Sheet response to Southern Hemisphere atmospheric and oceanic warming between 18 and 17 ka
Source: Sci Rep. 2019 Mar 11;9:4133. doi: 10.1038/s41598-019-39750-w (PMC6411896; doi:10.1038/s41598-019-39750-w)
Supplement: Supplementary file 1 — Supplementary Information [file 41598_2019_39750_MOESM1_ESM.pdf]

Supplementary Information

Phased Patagonian Ice Sheet response to Southern Hemisphere atmospheric and oceanic warming between 18 and 17 ka

Jacob M. Bendle[1*] Adrian P. Palmer[1] Varyl R. Thorndycraft[1] Ian P. Matthews[1]

[1]Centre for Quaternary Research, Geography Department, Royal Holloway, University of London, Egham, Surrey, TW20 0EX, UK

*Jacob.Bendle@rhul.ac.uk

S1. Site details

S1.1. The Lago General Carrera–Buenos Aires ice lobe

The Lago General Carrera–Buenos Aires (LGC–BA) basin of central Patagonia has been periodically occupied by a major ice lobe of the former Patagonian Ice Sheet (PIS). Over the last 1.0 Ma, the LGC–BA ice lobe has experienced at least 4 major advances (Singer et al., 2004; Kaplan et al., 2004, 2005; Douglass et al., 2006; Smedley et al., 2016; Hein et al., 2017). During full glacial episodes, the LGC–BA ice lobe advanced from a regional ice-divide over the Patagonian Andes (Glasser et al., 2005) to the Argentinean steppe around 150-200 km further east (Caldenius, 1932). The last major advance of the LGC–BA ice lobe occurred during the global Last Glacial Maximum (LGM; Kaplan et al., 2004; Douglass et al., 2006). Landform evidence (Glasser et al., 2005) and numerical ice sheet simulations (Hubbard et al., 2005) suggest that at the LGM, the LGC–BA lobe was a topographically-controlled, fast-flowing outlet glacier that had a major influence on regional ice discharge patterns, the location of ice-divides, and ice-surface profile.

During the last deglaciation, a proglacial lake developed around the retreating margins of the LGC–BA ice lobe, as evidenced by widespread relict shorelines and raised deltas (Turner et al., 2005; Bell et al., 2008; Glasser et al., 2016; Thorndycraft et al., 2019). Geomorphological and dating evidence suggest a complex glacial lake history with multiple lake level stages controlled by the long-term patterns of ice-retreat and the opening/closing of lake spillways (Glasser et al., 2016; Thorndycraft et al., 2019). The varved lake sediments reported in the main paper are associated with the earliest stages of lake evolution, and the onset of regional deglaciation (Bendle et al., 2017a).

S1.2. The Río Fenix Chico valley in eastern LGC–BA

The presence of varved lake sediments in the LGC–BA basin was first proposed by Caldenius (1932) in the early 20th century. Caldenius (1932), and later Sylwan (1989), observed laminated silts and clays in outcrops within the Río Fenix Chico valley, a narrow, steep-sided gorge incised through unconsolidated glaciolacustrine deposits (Fig. S1). These sediments were re-investigated by Bendle et al. (2017a) and directly dated for the first time, providing the first annually-resolved and calendar-year

chronology for PIS dynamics following the end of the LGM, and permitting the current study.

The varved deposits are banked against the innermost moraine of the LGM Fenix complex (Kaplan et al., 2004; Douglass et al., 2006; Smedley et al., 2016; Bendle et al., 2017a), namely the Fenix I moraine, which has a weighted mean age and standard deviation of 18.7 ± 1.7 ka (calculated using the recalibrated ^{10}Be dataset of Kaplan et al., 2011) based on cosmogenic nuclide exposure dating of moraine boulders. Using a Bayesian age model that integrated published boulder ages with calendar-year time-intervals defined by the FCMC17, Bendle et al. (2017a) refined the Fenix I age estimate, modelling a ~700-year period of glacier stability between 18,778 ± 615 and 18,086 ± 214 cal yr BP.

Varved sediments began to accumulate as the LGC–BA ice lobe retreated from the Fenix I moraine (Caldenius, 1932; Bendle et al., 2017a) and an ice-contact glacial lake developed (Glasser et al., 2016; Thorndycraft et al., 2019). ~2.0 km west of the Fenix I moraine, a glacier stillstand deposited the Menucos moraine. Only the crest of this moraine outcrops above the lacustrine sediments, which have part-buried the ridge (Bendle et al., 2017a). This younger limit has a weighted mean age of 17.5 ± 1.3 ka (cf. Kaplan et al., 2011). The Bayesian modelled age for Menucos moraine deposition is 17,710 ± 116 cal yr BP (Bendle et al. 2017a), within the uncertainties of boulder ages. Lithostratigraphic evidence supports a period of ice-proximal fan construction at Estancia Santa Maria (Fig. S3), ~4.0 km west of the Menucos moraine (Bendle et al., 2017a). This limit has not been directly dated (e.g. using cosmogenic nuclide exposure dating, or optically stimulated luminescence), but has a modelled age of 17,322 ± 116 cal yr BP (Bendle et al., 2017a). 6-9 km west of the Estancia Santa Maria limit, road cuttings reveal sediment sequences characteristic of ice-proximal subaqueous moraine deposition (Fig. S3), and represent a final position of relative ice-margin stability in the eastern end of LGC–BA (Bendle et al., 2017a).

S2. Construction of the varve chronology

S2.1. Varve structure

Bendle et al. (2017a) detail the sedimentology of the laminated deposits, and present criteria for the interpretation of varve structure, however, the key criteria are summarised here. The regular alternation of coarse and fine laminations is typical of glaciolacustrine varves observed at the macro- (Ashley, 1975; Smith and Ashley, 1985; Ridge et al., 2012) and microscale (Ringberg and Erlström, 1999; Palmer et al., 2010, 2012; MacLeod et al., 2011). Specifically, the deposits comprise regular alternations of texturally distinct layers: (i) coarse (silt and/or very fine sand) layers that are internally complex, and contain multiple massive or graded laminations; and (ii) fine layers that grade upwards from very-fine silt to clay. The coarse component forms in the melt season (spring/summer) as meltwater plumes transport sediment to the lake; and the fine component settles from suspension during the quiescent non-melt season (autumn/winter), possibly under lake ice-cover (cf. Ashley, 1975; Ringberg and Erlström, 1999; Palmer et al., 2010, 2012; Ridge et al., 2012).

S2.2. Developing the composite FCMC17

The composite Fenix Chico Master Varve Chronology 2017 (FCMC17) is constructed from local varve chronologies at five sites in the Río Fenix Chico valley (Fig. S1 and S3; Bendle et al., 2017a). Each sequence contains common sedimentary marker layers that allow correlation between records, and the evaluation of potential gaps in site varve series (Fig. S4). Cross-dating (i.e. the correlation of site varve series; Lamoureux, 2001) was performed using standardised varve thickness data (e.g. Heideman et al., 2015) to account for systematic varve thickness differences associated with the relative proximity of each site to the former LGC–BA ice-margin. The cross-dating process yields high visual (Fig. S4) and statistical correlations (Table S1) between varve series, and a composite record of 994 ± 36 varve years (vyrs) minimum duration. While most breaks in site varve series were bridged using better preserved sequences, some breaks remain in the FCMC17, however, these are likely to be of minor duration, and therefore insignificant for analysis of the overall patterns of glacier evolution (Bendle et al., 2017a).

S2.3. Ho tephra identification and absolute dating of the FCMC17

The LGC–BA varve record has been anchored to the calendar-year timescale through geochemical identification of the Ho tephra layer (Weller et al., 2014), using major-

element (WDS-EPMA) and trace-element (XRF) analyses (Bendle et al., 2017a; Fig. S5). The tephra layer provides an independent marker layer for the correlation of site varve sequences, and is deposited in vyr 605 ± 27 in the composite chronology (Fig. S2a). Using eight published radiocarbon ages (Miranda et al., 2013; Table S2) the age of the Ho tephra was recalibrated to 17,378 ± 118 cal a BP using a phase model in Oxcal v4.3 (Bronk Ramsey, 2009; Fig. S2d) and the SHCal13 calibration curve (Hogg et al., 2013). Calendar-year ages were extrapolated from vyr 605 ± 27 across the remaining varves. This constrains the varve record between 17,997 ± 145 and 16,982 ± 129 cal yr BP (Bendle et al., 2017a).

S3. Determining ice lobe retreat rates

Fig. S1 shows the location of varve sites in the Río Fenix Chico valley. The position of the sites allows intervals of the composite FCMC17 record to be attributed to discrete phases of ice-margin retreat, and average retreat rates calculated based on the distances between former ice-margin positions (Fig. S1 and S2).

S3.1. Retreat phase 1:

Varves formed at FC-B and FC-C/D (Fig. S1) are attributed to ~2.0 km ice-margin retreat from the late-LGM Fenix I moraine (Kaplan et al., 2004; Douglass et al., 2006) to the Menucos moraine (*Phase 1*). This interpretation is confirmed by a lack of correlative varves in sites located to the west of the Menucos moraine. This phase comprises 210 ± 16 vyrs and yields a mean retreat rate of 9.52 m yr^{-1}. As we were unable to record the basal varves at FC-B and FC-C/D the 210 ± 16 vyrs estimated for *Retreat phase 1* represent a minimum duration. Thus, 'real-world' retreat rates may have been slightly lower than 9.52 m-yr^{-1}.

The stable multi-decadal varve thickness trend in *Phase 1* (Fig. S4) likely reflects a slow retreat or a glacier stillstand (Leonard, 1997). The increasing varve thickness trend observed over the final ~60 years of *Phase 1* has previously been inferred to reflect increasing ablation rates and meltwater/sediment fluxes (Bendle et al., 2017) during this period. While increasing varve thickness could also be interpreted to represent a glacier readvance signal, there is no stratigraphic evidence (e.g.

glaciotectonised varves) in support of this alternative scenario (Fig. S2; see Bendle et al., 2017).

S3.2. Retreat phase 2:

The onset of varve sedimentation at sites FC-F and FC-F1 (Fig. S1) records ice-margin retreat from the Menucos moraine, and the onset of *Phase 2*, which lasted 389 ± 9 vyrs. An abrupt 10-year varve thickness increase at the start of *Retreat phase 2* is interpreted to reflect increasing meltwater discharges characteristic of the initial decades of glacier retreat (e.g. Leonard, 1997; Larsen et al., 2011), and suggests that varves started to accumulate almost immediately behind the ice-contact face of the Menucos moraine. We infer that the next ice-margin position was established at, or near, the Santa Maria ice-proximal fan complex (Bendle et al., 2017a), ~4.5 km west of the Menucos moraine (Fig. S1 and S2). Within this period, the trend in varve thickness declines persistently to the Ho tephra layer dated to 17,378 ± 118 cal yr BP, which is taken as an approximate end date for *Phase 2*. This assumption yields an ice-margin retreat rate of 11.56 m-yr^{-1}, ~21% faster than in *Phase 1*. The sustained decreasing thickness trend observed in this interval is consistent with a gradual reduction in glacier extent. Bendle et al. (2017a) report frequent coarse-grained sediment influxes (e.g. graded sand beds) in this interval, suggesting that an ice-margin position at this location is not improbable, given that such influxes are characteristics of ice-proximal deposition (Gustavson, 1975; Ashley, 1975). Nonetheless, we consider the inferred ~4.5 km retreat attributed to this phase a minimum retreat distance, and thus the calculated retreat rate could represent an underestimate.

S3.3. Retreat phase 3:

An abrupt 10-year increase in varve thickness that approximately coincides with deposition of the Ho tephra (17,378 ± 118 cal yr BP) marks the onset of enhanced ablation (Leonard, 1997) at the beginning of *Phase 3*, and more extensive glacier recession from the Río Fenix Chico valley. Varve thickness decreases rapidly over the next 395 ± 9 vyrs, until our varve record ends at 16,982 ± 127 cal yr BP. We suggest that persistent ice-rafted debris (IRD) from ~17,145 ± 122 cal yr BP reflects the development of a calving ice-front (e.g. Larsen et al., 2015), and retreat of the LGC-

BA ice lobe into deeper lake waters by this time. The precise ice-margin position(s) established during *Phase 3* cannot yet be definitively confirmed. However, the subaqueous moraine sequences found in road cuttings ~6.0-9.0 km west of the Santa Maria limit provide a probable point of glacier stabilisation (Fig. S3). Using this limit, a mean retreat rate of 18.99 m-yr^{-1} is calculated, ~64% faster than in *Phase 2*. Again, we consider this a minimum retreat distance, and thus the calculated retreat rate could represent an underestimate.

S4. References

1. Ashley, G.M. 1975. Rhythmic sedimentation in Glacial Lake Hitchcock, Massachusetts-Connecticut, in: Jopling, A.V., McDonald, B.C. (Eds.) Society of Economic Paleontologists and Mineralogists Special Publication, 23, pp. 304–320.
2. Bell, C.M. 2008. Punctuated drainage of an ice-dammed Quaternary lake in Southern South America. Geografiska Annaler: Series A Physical Geography, 90, 1–17.
3. Bendle, J.M., Palmer, A.P., Thorndycraft, V.R., Matthews, I.P. 2017a. High-resolution chronology for deglaciation of the Patagonian Ice Sheet at Lago Buenos Aires (46.5°S) revealed through varve chronology and Bayesian age modelling. Quaternary Science Reviews, 177, 314–339.
4. Bendle, J.M., Thorndycraft, V.R.., Palmer, A.P., 2017b. The glacial geomorphology of the Lago Buenos Aires and Lago Pueyrredón ice lobes of central Patagonia. Journal of Maps, 13, 654–673.
5. Bronk Ramsey, C. 2009. Bayesian analysis of radiocarbon dates. Radiocarbon, 51, 337–360.
6. Douglass, D.C., Singer, B.S., Kaplan, M.R., Mickelson, D.M. & Caffee, M.W. 2006. Cosmogenic nuclide surface exposure dating of boulders on last-glacial and late-glacial moraines, Lago Buenos Aires, Argentina: interpretive strategies and paleoclimate implications. Quaternary Geochronology, 1, 43–58.
7. Douglass, D.C., Singer, B.S., Kaplan, M.R., Mickelson, D.M. & Caffee, M.W. 2006. Cosmogenic nuclide surface exposure dating of boulders on last-glacial and late-glacial moraines, Lago Buenos Aires, Argentina: interpretive strategies and paleoclimate implications. Quaternary Geochronology, 1, 43–58.
8. Glasser, N.F. & Jansson, K.N. 2005. Fast-flowing outlet glaciers of the last glacial maximum Patagonian Icefield. Quaternary Research, 63, 206–211.
9. Glasser, N.F., Jansson, K.N., Duller, G.A., Singarayer, J., Holloway, M. & Harrison, S. 2016. Glacial lake drainage in Patagonia (13-8 kyr) and response of the adjacent Pacific Ocean. Scientific Reports, 6. 21064.
10. Gustavson, T.C. 1975. Bathymetry and sediment distribution in proglacial

Malaspina Lake, Alaska. Journal of Sedimentary Petrology, 45, 450–461.
11. Hein, A.S., Cogez, A., Darvill, C.M., Mendelova, M., Kaplan, M.R., Herman, F., Dunai, T.J., Norton, K., Xu, S., Christl, M. & Rodés, Á. 2017. Regional mid-Pleistocene glaciation in central Patagonia. Quaternary Science Reviews, 164, 77–94.
12. Hogg, A.G., Hua, Q., Blackwell, P.G., Niu, M., Buck, C.E., Guilderson, T.P., Heaton, T.J., Palmer, J.G., Reimer, P.J., Reimer, R.W. & Turney, C.S. 2013. SHCal13 Southern Hemisphere calibration, 0–50,000 years cal BP. Radiocarbon, 55, 1–15.
13. Hubbard, A., Hein, A.S., Kaplan, M.R., Hulton, N.R. & Glasser, N. 2005. A modelling reconstruction of the last glacial maximum ice sheet and its deglaciation in the vicinity of the Northern Patagonian Icefield, South America. Geografiska Annaler: Series A Physical Geography, 87, 375–391.
14. Kaplan, M.R., Ackert, R.P., Singer, B.S., Douglass, D.C. & Kurz, M.D. 2004. Cosmogenic nuclide chronology of millennial-scale glacial advances during O-isotope stage 2 in Patagonia. Geological Society of America Bulletin, 116, 308–321.
15. Kaplan, M.R., Ackert, R.P., Singer, B.S., Douglass, D.C. & Kurz, M.D. 2004. Cosmogenic nuclide chronology of millennial-scale glacial advances during O-isotope stage 2 in Patagonia. Geological Society of America Bulletin, 116, 308–321.
16. Kaplan, M.R., Douglass, D.C., Singer, B.S., Ackert, R.P. & Caffee, M.W. 2005. Cosmogenic nuclide chronology of pre-last glacial maximum moraines at Lago Buenos Aires, 46°S, Argentina. Quaternary Research, 63, 301–315.
17. Kaplan, M.R., Strelin, J.A., Schaefer, J.M., Denton, G.H., Finkel, R.C., Schwartz, R., Putnam, A.E., Vandergoes, M.J., Goehring, B.M. & Travis, S.G. 2011. In-situ cosmogenic ^{10}Be production rate at Lago Argentino, Patagonia: implications for late-glacial climate chronology. Earth and Planetary Science Letters, 309, 21–32.
18. Larsen, D.J., Geirsdóttir, Á. & Miller, G.H. 2015. Precise chronology of Little Ice Age expansion and repetitive surges of Langjökull, central Iceland. Geology, 43, 167-170.
19. Larsen, D.J., Miller, G.H., Geirsdóttir, Á. & Thordarson, T. 2011. A 3000-year varved record of glacier activity and climate change from the proglacial lake Hvítárvatn, Iceland. Quaternary Science Reviews, 30, 2715–2731.
20. Leonard, E.M. 1997. The relationship between glacial activity and sediment production: evidence from a 4450-year varve record of neoglacial sedimentation in Hector Lake, Alberta, Canada. Journal of Paleolimnology, 17, 319–330.
21. MacLeod, A., Palmer, A., Lowe, J., Rose, J., Bryant, C. & Merritt, J. 2011. Timing of glacier response to Younger Dryas climatic cooling in Scotland. Global and Planetary Change, 79, 264–274.

22. Miranda, C.G., Moreno, P.I., Vilanova, I. & Villa-Martinez, R.P. 2013. Glacial fluctuations in the Coyhaique-Balmaceda sector of central Patagonia (45°S-46°S) during the last glacial termination. Bollettino Geofisica Teorica Applicata, 54, 268–271.
23. Ólafsdóttir, K.B., Geirsdóttir, Á., Miller, G.H. & Larsen, D.J. 2013. Evolution of NAO and AMO strength and cyclicity derived from a 3-ka varve-thickness record from Iceland. Quaternary Science Reviews, 69, 142–154.
24. Palmer, A.P., Rose, J. & Rasmussen, S.O. 2012. Evidence for phase-locked changes in climate between Scotland and Greenland during GS-1 (Younger Dryas) using micromorphology of glaciolacustrine varves from Glen Roy. Quaternary Science Reviews, 36, 114–123.
25. Palmer, A.P., Rose, J., Lowe, J.J. & MacLeod, A. 2010. Annually resolved events of Younger Dryas glaciation in Lochaber (Glen Roy and Glen Spean), western Scottish Highlands. Journal of Quaternary Science, 25, 581–596.
26. Ridge, J.C., Balco, G., Bayless, R.L, Beck, C.C, Carter, L..B, Dean, J.L, Voytek, E.B & Wei, J.H., 2012. The new North American Varve Chronology: A precise record of southeastern Laurentide Ice Sheet deglaciation and climate, 18.2-12.5 kyr BP, and correlations with Greenland ice core records. American Journal of Science, 312, 685–722.
27. Ringberg, B. & Erlström, M. 1999. Micromorphology and petrography of Late Weichselian glaciolacustrine varves in southeastern Sweden. Catena 35, 147–177.
28. Singer, B.S., Ackert, R.P. & Guillou, H. 2004. $^{40}Ar/^{39}Ar$ and K-Ar chronology of Pleistocene glaciations in Patagonia. Geological Society of America Bulletin, 116, 434–450.
29. Smedley, R.K., Glasser, N.F. & Duller, G.A.T. 2016. Luminescence dating of glacial advances at Lago Buenos Aires (~46°S), Patagonia. Quaternary Science Reviews, 134, 59—73.
30. Smith, N.D. & Ashley, G.M., 1985. Proglacial lacustrine environment. In Glacial Sedimentary Environments, Ashley G.M., Shaw J., Smith, N.D. (Eds) Society of Palaeontologists and Mineralogists: Tulsa, OK, 135–212.
31. Stern, C., Porras, M. & Maldonado, A. 2015. Tephrochronology of the upper Río Cisnes valley (44°S), southern Chile. Andean Geology, 42, 173–189.
32. Thomas, E.K. & Briner, J.P., 2009. Climate of the past millennium inferred from varved proglacial lake sediments on northeast Baffin Island, Arctic Canada. Journal of Paleolimnology, 41, 209–224.
33. Thorndycraft, V.R., Bendle, J.M., Benito, G., Davies, B.J., Sancho, C., Palmer, A.P., Fabel, D., Medialdea, A. and Martin, J.R.V. 2019. Glacial lake evolution and Atlantic-Pacific drainage reversals during deglaciation of the Patagonian Ice Sheet. Quaternary Science Reviews, 203, 102–127.
34. Turner, K.J., Fogwill, C.J., McCulloch, R.D. & Sugden, D.E. 2005. Deglaciation of the eastern flank of the north patagonian icefield and associated continental-

scale lake diversions. Geografiska Annaler: Series A Physical Geography, 87, 363–374.

35. Weller, D., Miranda, C.G., Moreno, P.I., Villa-Martínez, R. & Stern, C.R. 2014. The large late-glacial Ho eruption of the Hudson volcano, southern Chile. Bulletin of Volcanology, 76, 831.
36. Weller, D.J., Miranda, C.G., Moreno, P.I., Villa-Martínez, R. & Stern, C.R. 2015. Tephrochronology of the southernmost Andean southern volcanic zone, Chile. Bulletin of Volcanology, 77, 107.

Figures:

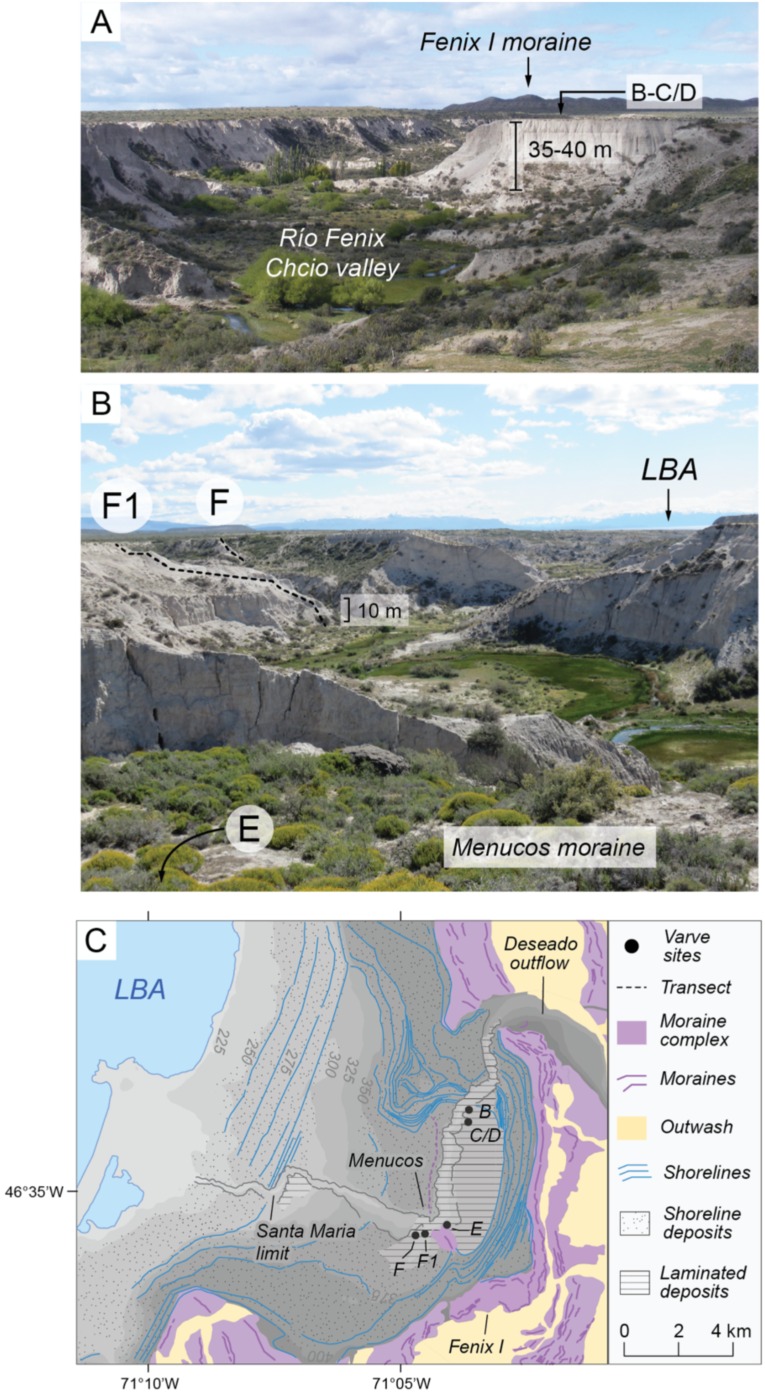

Fig. S1. Field photographs of Río Fenix Chico valley and location of varve sites. (A) View north-east along the Río Fenix Chico gorge, taken from the Menucos moraine. Approximate position of FC-B and FC-C/D sites indicated. (B) View west along Río Fenix Chico valley. Photo taken from crest of part-buried Menucos moraine, showing sites FC-F and FC-F1 and present-day Lago Buenos Aires ~9 km down valley. (C) Geomorphological context of the Río Fenix Chico valley (cf. Bendle et al., 2017b) and location of varve sites in relation to former ice-margin limits.

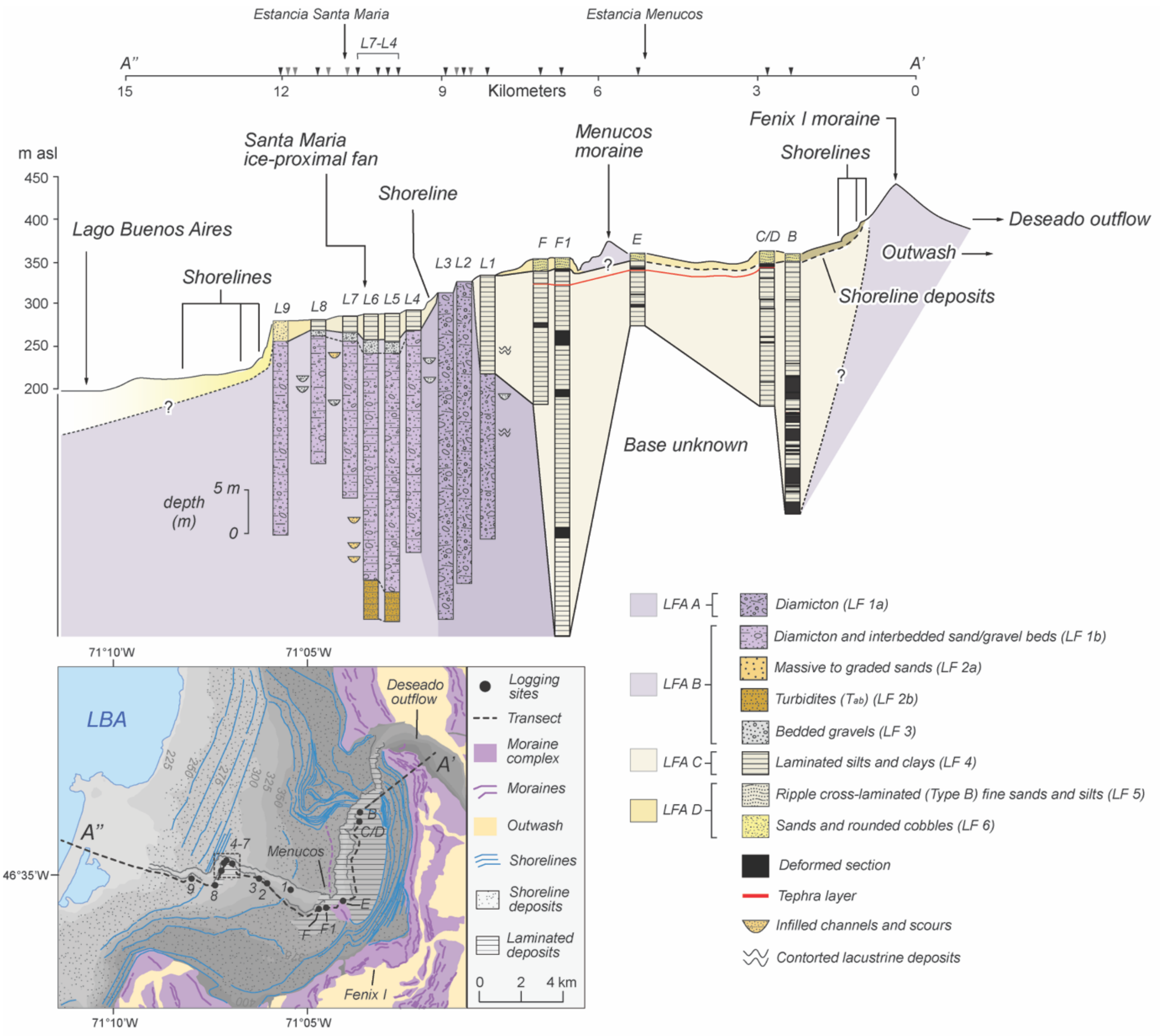

Fig. S2. Lithostratigraphy of the Río Fenix Chico valley cf. Bendle et al. (2017a). The location of logging sites (including varve sites) in shown in geomorphological map. Varve sites B and C/D are positioned between the Fenix I and Menucos moraines, offering constraint on the duration of Retreat phase 1. Sites F and F1 are positioned between the Menucos moraine and Santa Maria ice-proximal fan, offering constraint on the duration of Retreat phase 2. Site E overlies the ice-distal flank of part-buried Menucos moraine, and contains mm-scale varves that record Retreat phase 3.

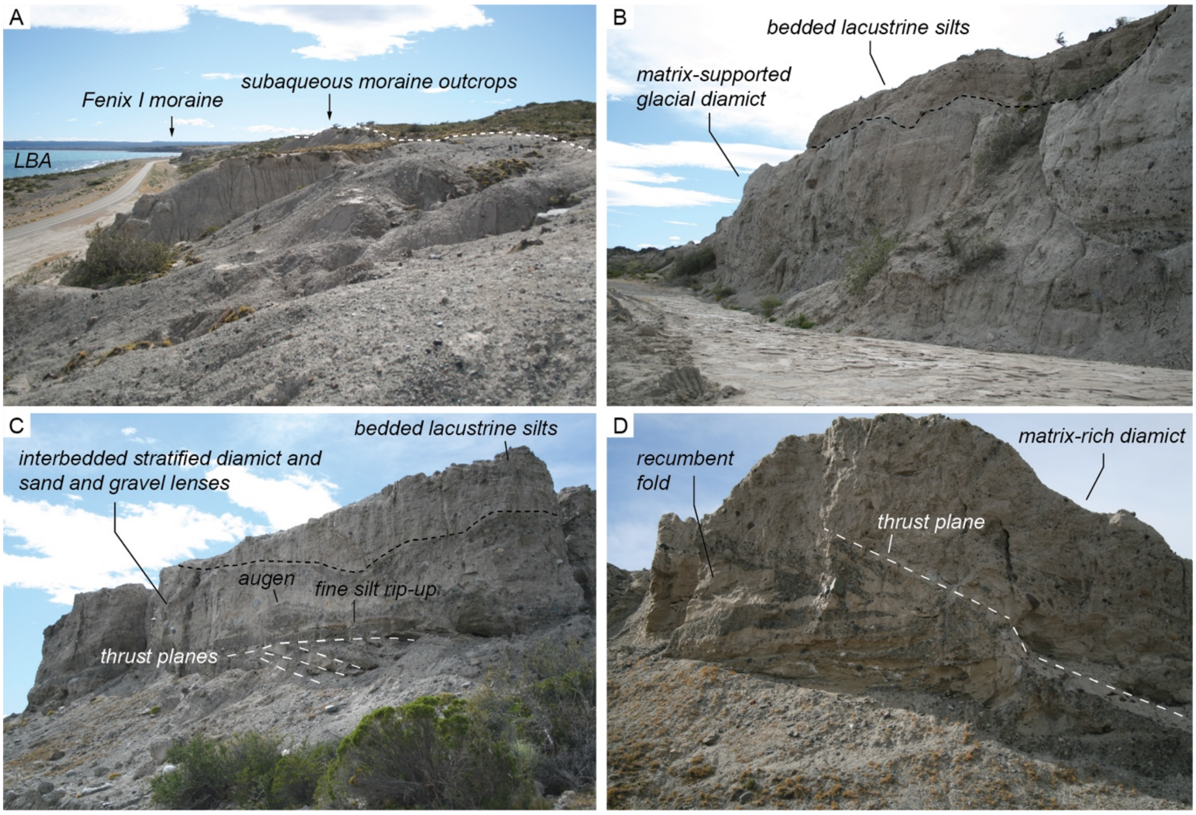

Fig. S3. Road sections between ~6-9 km west of the Santa Maria limit, displaying evidence of ice-proximal subaqueous moraine deposition (e.g. Benn, 1996; Evans et al., 2012). (A) View north-east showing moraine outcrops in foreground and the Fenix I moraine crest ~12-15 km further east. (B) Matrix-supported, and crudely stratified glacial diamict, overlain by fine-grained laminated silts. (C) Crudely-stratified diamict lenses interbedded with moderately-sorted sand and gravel clinoforms, and overlain by fine-grained lacustrine silts. Lower part of outcrop has been glaciotectonized (e.g. see thrust planes, rip-up intraclasts, folding, and augens). (D) Recumbent fold and thrust planes in interbedded diamict and sand and gravel lenses, typical of glacial overriding (Evans et al., 2012).

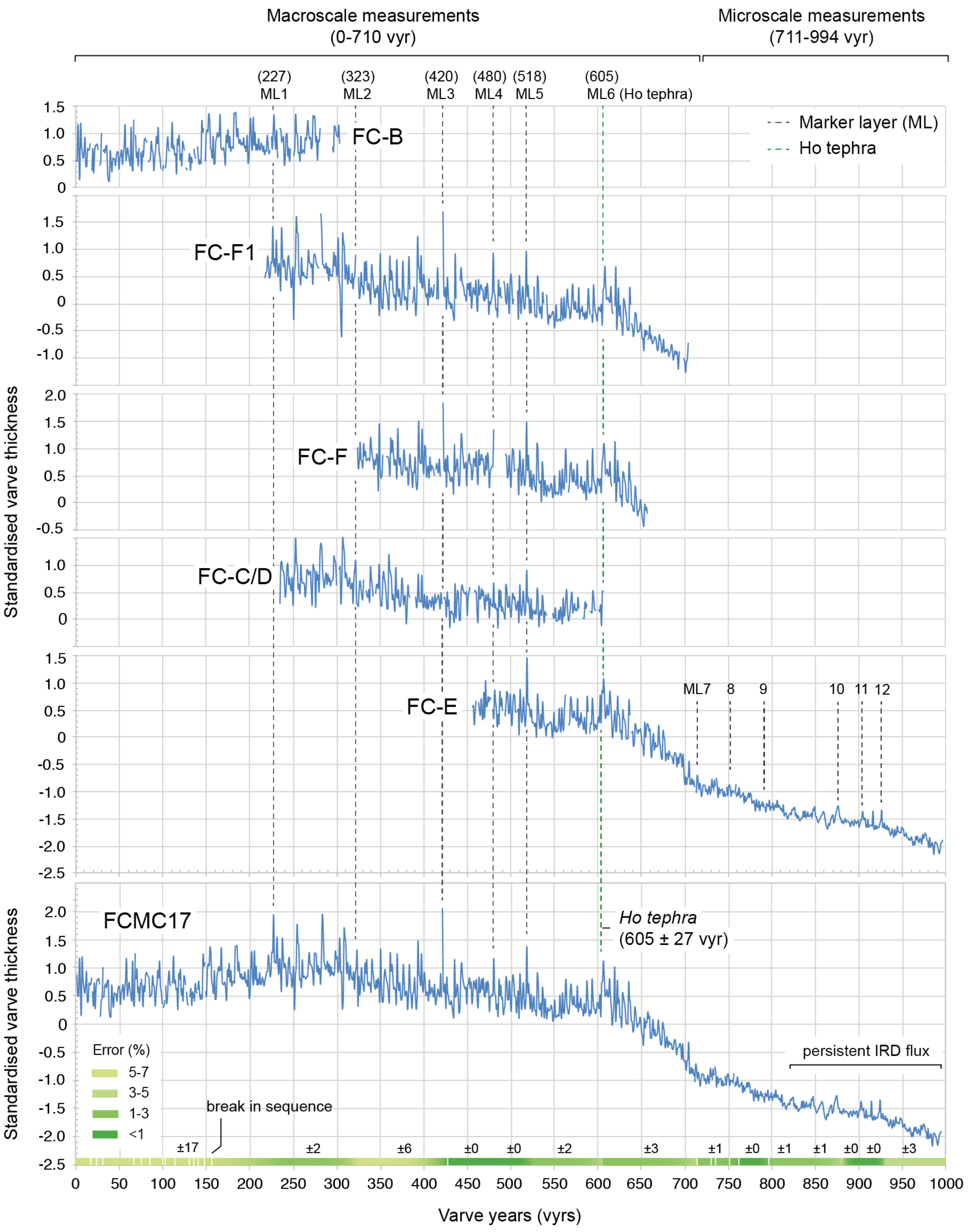

Fig. S4. Correlation of site varve series and construction of the composite Fenix Chico Master Varve Chronology 2017 (FCMC17; Bendle et al., 2017a). Correlations were made using sedimentary marker layers and varve thickness trends. Gaps in site varve series represent sections with 'missing' or disturbed varves, which were mostly bridged through cross-dating. Counting uncertainties are attributed to counting intervals, which were defined by the presence of marker layers 1-12 (e.g. ML1 to ML2 represents one counting interval). Minor breaks in the FCMC17 record, which could not be bridged by cross-dating, are also indicated.

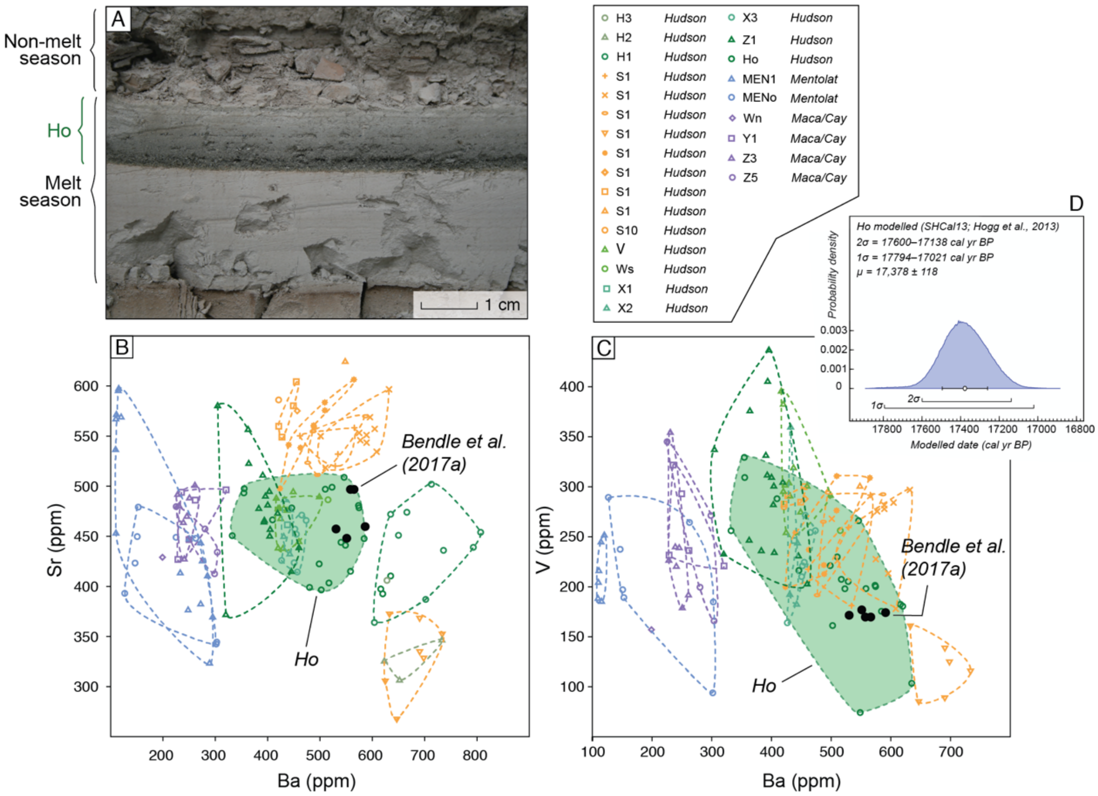

Fig. S5. (A) Field image of Ho tephra layer observed in the upper portion of a melt-season layer at site FC-E. (B) Ba vs. Sr. and (C) Ba vs. V. trace-element plots showing correlation with the Ho tephra (cf. Weller et al., 2015). Published trace element data (derived from Weller et al., 2014; 2015; Stern et al., 2015) are grouped according to the descriptive tephra zones presented in Weller et al. (2015). (D) Remodelled Ho tephra age based on radiocarbon determinations of Miranda et al. (2013) and SHCal13 calibration curve (Hogg et al., 2013).

Tables:

Table S1. Pearson's correlation matrix of standardised varve thickness for correlated varves between five sites. Degrees of freedom (*n*-2) reported in parentheses. Significance (*p*-value) = <0.0001 (two-tailed) for all correlations.

	FC-F1	FC-F	FC-E	FC-C/D	FC-B
FC/F1	1				
FC-F	0.96 (292)	1			
FC-E	0.95 (223)	0.90 (174)	1		
FC-C/D	0.89 (315)	0.86 (229)	0.84 (120)	1	
FC-B	0.71 (64)	-	-	0.69 (49)	1

Table S2. Radiocarbon determinations for organic material sampled from above (<Ho; 'younger than') and below (>Ho; 'older than') the Ho tephra layer at four sites, cf. Miranda et al. (2013). Recalibrated cal a BP ages (2-sigma) determined using the SHCal13 cf. Hogg et al. (2013) calibration curve.

Stratigraphy	Laboratory code	^{14}C a BP	cal a BP
<Ho	UCIAMS-122978	13,430 ± 50	16,170 ± 101
<Ho	CAMS-159614	13,720 ± 45	16,499 ± 121
<Ho	CAMS-159606	13,810 ± 110	16, 649 ± 190
<Ho	CAMS-159607	14,220 ± 45	17,222 ± 96
>Ho	CAMS-159613	14,345 ± 45	17,490 ± 90
>Ho	UCIAMS-122999	14,670 ± 45	17,801 ± 87
>Ho	CAMS-154860	14,735 ± 30	17,865 ± 78
>Ho	UCIAMS-123030	14,800 ± 90	17,895 ± 115
